# Supplementary material for: Prediction of risk of acquiring urinary tract infection during hospital stay based on machine-learning: A retrospective cohort study
Source: PLoS One. 2021 Mar 31;16(3):e0248636. doi: 10.1371/journal.pone.0248636 (PMC8011767; doi:10.1371/journal.pone.0248636)
Supplement: S1 Table — (PDF) [file pone.0248636.s001.pdf]

S1 Table. HAIR definitions of UTI.

CDC criteria[1]	HAIR definition[2]
• A urine culture of $\geq 10^5$ cfu/mL plus one or more of the following clinical criteria: temperature > 38^0C, frequency, urgency, dysuria or suprapubic tenderness.	• Culture of $\geq 10^5$ cfu/mL of a dominating pathogen in urine culture *plus* UTI-relevant[3] antibiotic treatment<br>• A urine culture of a single pathogen of $\geq 10^4$ cfu/mL *plus* UTI-relevant antibiotic treatment.
• At least two of the aforementioned clinical criteria: positive dipstick for leukocytes and/or nitrate, physicians' clinical UTI diagnosis and prescription of antibiotic treatment.	• Treatment with a UTI-specific[3] antibiotic prescribed without the presence of a positive urine culture.

[1] Adapted for use in point prevalence studies in Denmark: Definition and coding of nosocomial infections: Statens Serum Institut; 1997.

[2] Leth RA, Møller JK. Surveillance of hospital-acquired infections based on electronic hospital registries. J Hosp Infect. 2006;82:71-9.

[3] Antibiotics used in general for treatment of urinary tract infections or blood stream infections.

[4] Orally prescribed antibiotics (e.g. pivmecillinam, trimethoprim, nitrofurantoin, sulfamethizole) which are solely prescribed in cases of urinary tract infection in Denmark.
