# Supplementary material for: Prediction of risk of acquiring urinary tract infection during hospital stay based on machine-learning: A retrospective cohort study
Source: PLoS One. 2021 Mar 31;16(3):e0248636. doi: 10.1371/journal.pone.0248636 (PMC8011767; doi:10.1371/journal.pone.0248636)
Supplement: S2 Table — (PDF) [file pone.0248636.s002.pdf]

S2 Table. Test characteristics of the UTI prediction models

Model Description	Average Squared Error		ROC Index	
	Validation data	Test data	Validation data	Test data
**Entry model**				
Neural Network	0.051851	0.053789	0.833	0.841
Gradient Boosting	0.052221	0.054687	0.828	0.833
Regression	0.052441	0.054970	0.828	0.833
DecTree -3WaySplit	0.052527	0.054563	0.820	0.829
Decision Tree	0.052617	0.054721	0.812	0.818
**HA-UTI model**				
Neural Network	0.045356	0.048920	0.777	0.770
Gradient Boosting	0.045787	0.049570	0.760	0.750
Regression	0.045887	0.049729	0.749	0.735
DecTree -3WaySplit	0.045912	0.049846	0.764	0.758
Decision Tree	0.046027	0.050034	0.736	0.709
