# Supplementary material for: Prediction of risk of acquiring urinary tract infection during hospital stay based on machine-learning: A retrospective cohort study
Source: PLoS One. 2021 Mar 31;16(3):e0248636. doi: 10.1371/journal.pone.0248636 (PMC8011767; doi:10.1371/journal.pone.0248636)
Supplement: S3 Table — (PDF) [file pone.0248636.s003.pdf]

S3 Table. The input (predictor) variables used in the Decision Tree machine-learning model for the UTI prediction for the HAI-UTI model.

**Input variable (at admission)**	**Number of splitting rules**	**Training Importance**	**Validation Importance**	**Ratio of validation to training importance**
Age	1	1.0000	1.0000	1.0000
CA-UTI during admission	3	0.4837	0.4631	0.9573
Trigger_05_IUC[1]	3	0.4411	0.3876	0.8786
Admitted_org_id_text	2	0.4002	0.2355	0.5886
Previous CA-UTI, admission	1	0.3712	0.3560	0.9591
OPCS CVC[3] time of insertion	2	0.3399	0. 3609	1.0619
Sex	4	0.3307	0.3458	1.0457
Previous HA-UTI, admission	1	0.1948	0.1635	0.8393
Admission_type	1	0.1916	0.1760	0.9186
Previous IUC[1], admission	2	0.1767	0.1710	0.9676
Admission_hospital_text	1	0.1497	0.1108	0.7404
ICD-10 Urinary retention	1	0.0803	0.1058	1.3177
ICD-10 Neurological disease	0	0.0000	0.0000	
Referral diagnosis	0	0.0000	0.0000	
OPCS CVC[2] replacement	0	0.0000	0.0000	
OPCS CVC[2] time of removal	0	0.0000	0.0000	
TRIGGER_203 Fever	0	0.0000	0.0000	
ICD-10 Unresolved spinal injury	0	0.0000	0.0000	
ICD-10 Atrial fibrillation	0	0.0000	0.0000	
ICD-10 Hypertension	0	0.0000	0.0000	
ICD-10 Chronic kidney disease	0	0.0000	0.0000	
ICD-10 Stroke	0	0.0000	0.0000	
ICD-10 Diabetes	0	0.0000	0.0000	
ICD-10 COPD[3]	0	0.0000	0.0000	
TRIGGER_06 permanent IUC[1]	0	0.0000	0.0000	
TRIGGER_303 Fever	0	0.0000	0.0000	
Readmission	0	0.0000	0.0000	
OPCS Bladder scan	0	0.0000	0.0000	
ICD-10 Unconsciousness	0	0.0000	0.0000	
TRIGGER_13 Bladder retention	0	0.0000	0.0000	

CA-UTI: Community Acquired UTI; HA-UTI: Hospital Acquired UTI.

[1]IUC: Indwelling urinary catheter

[2]Central venous catheter

[3]Chronic obstructive pulmonary disease

OPCS: operation and procedure codes
