# Supplementary figures and images for: Prediction of risk of acquiring urinary tract infection during hospital stay based on machine-learning: A retrospective cohort study
Source: PLoS One. 2021 Mar 31;16(3):e0248636. doi: 10.1371/journal.pone.0248636 (PMC8011767; doi:10.1371/journal.pone.0248636)

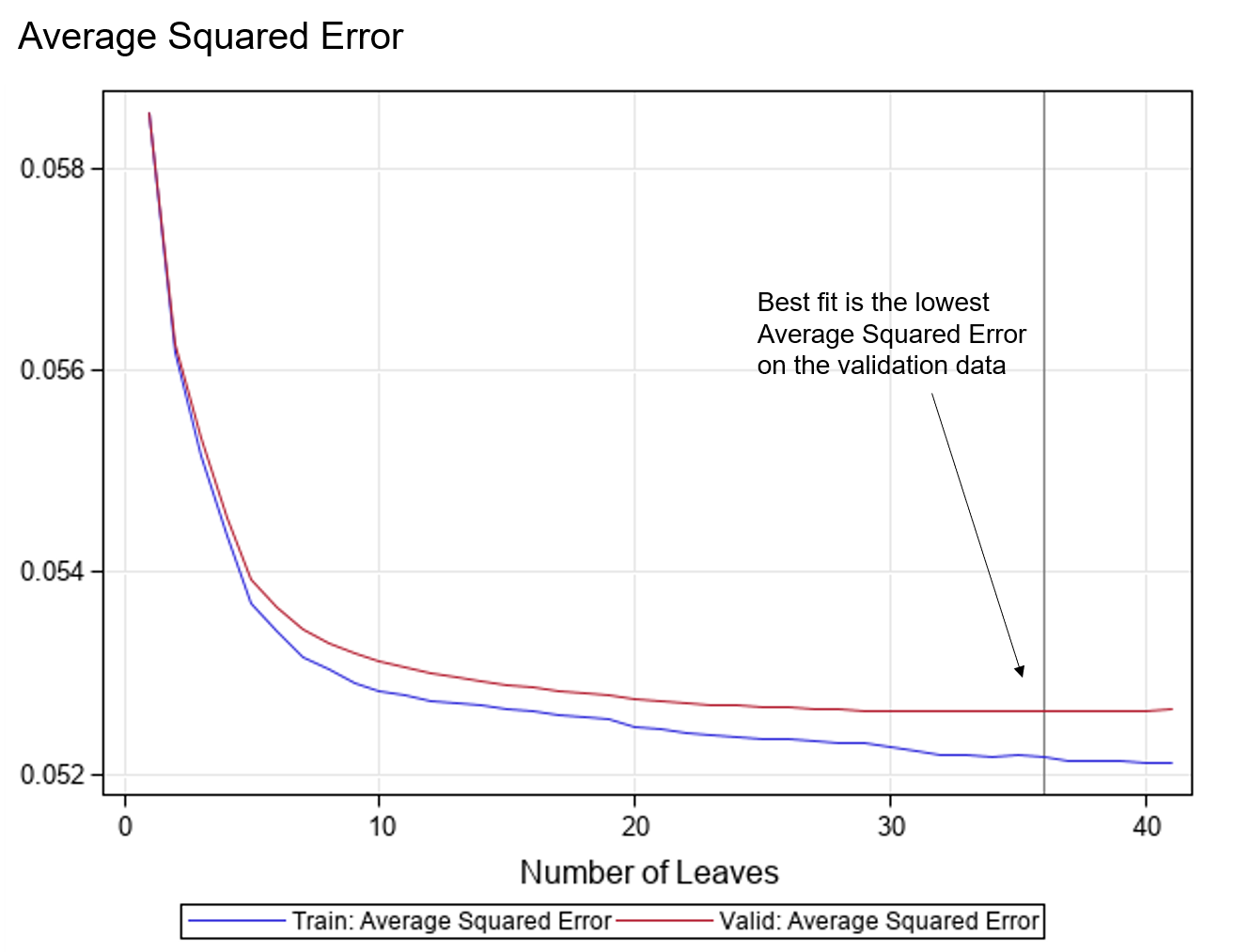

Supplement: S1 Fig — (TIF) [file pone.0248636.s004.tif]

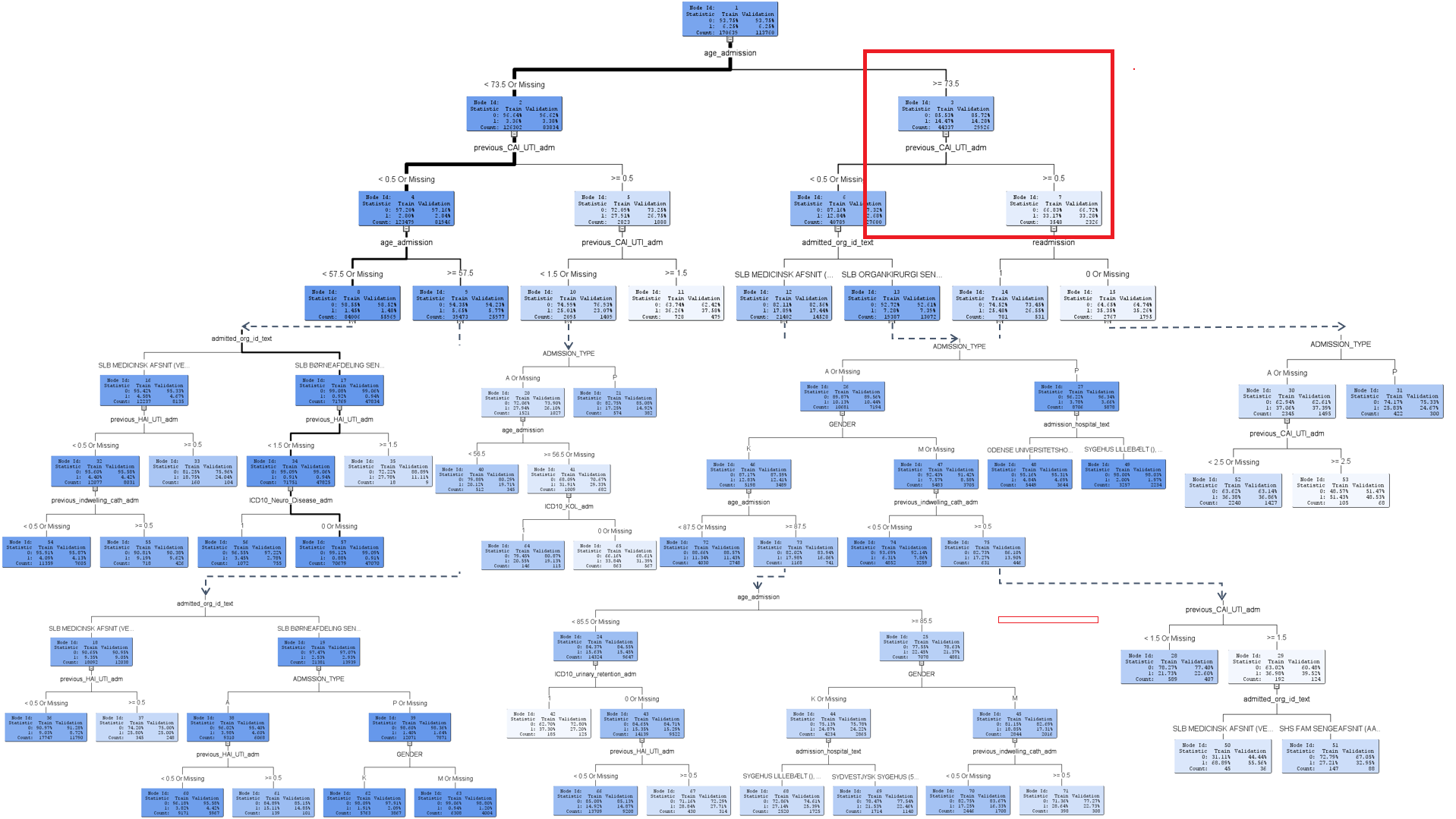

Supplement: S2 Fig — (TIF) [file pone.0248636.s005.tif]

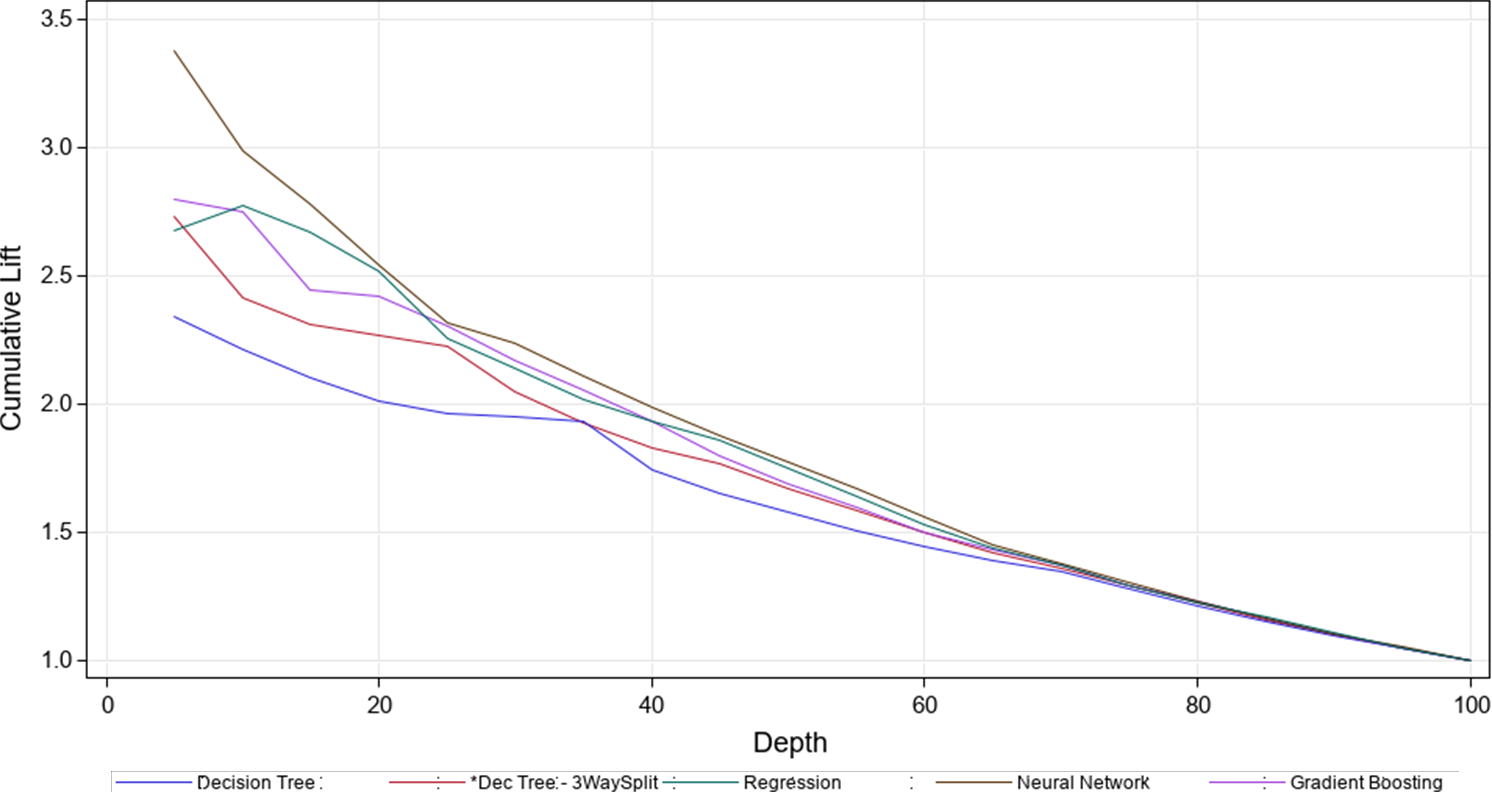

Supplement: S3 Fig — (TIF) [file pone.0248636.s006.tif]

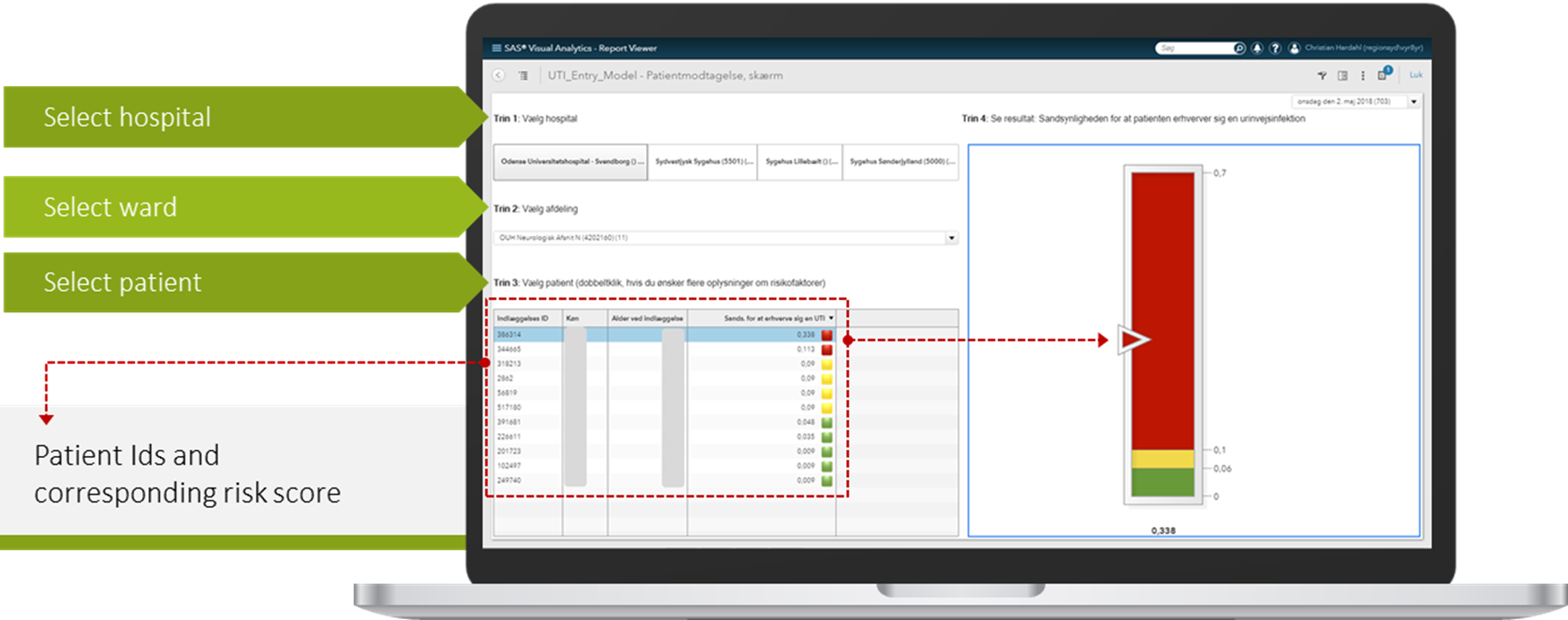

Supplement: S4 Fig — (TIF) [file pone.0248636.s007.tif]

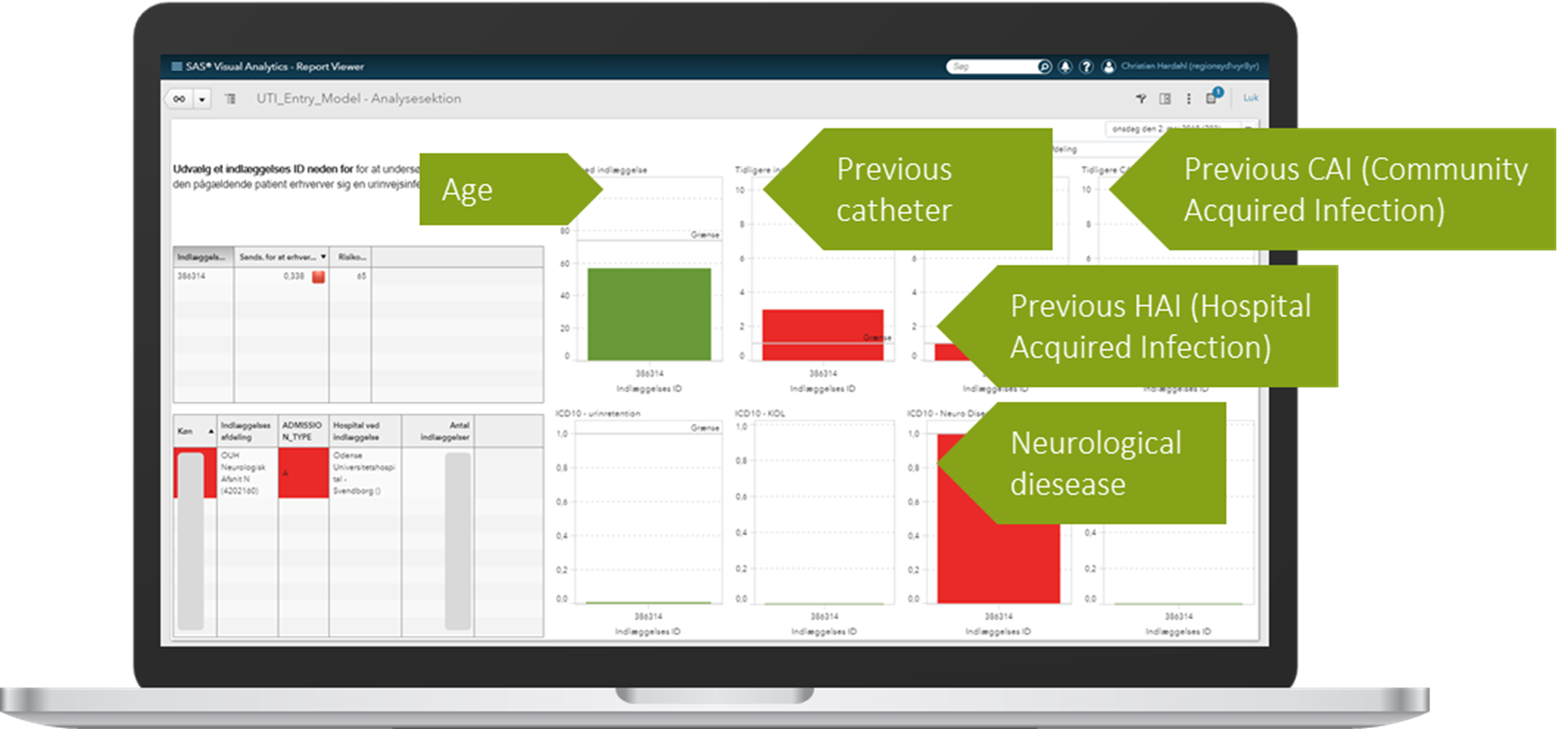

Supplement: S5 Fig — (TIF) [file pone.0248636.s008.tif]
