# Supplementary material for: Prediction of risk of acquiring urinary tract infection during hospital stay based on machine-learning: A retrospective cohort study
Source: PLoS One. 2021 Mar 31;16(3):e0248636. doi: 10.1371/journal.pone.0248636 (PMC8011767; doi:10.1371/journal.pone.0248636)
Supplement: S2 Appendix — (PDF) [file pone.0248636.s010.pdf]

S2 Appendix. SAS tools employed.

The tools used for the solution presented in the paper are **SAS® Content Categorization** (for text analytics), **SAS® Data Integration Studio** (for data integration/management), **SAS® Enterprise Miner™** (for predictive modelling), and **SAS® Visual Analytics** (for operationalizing the results).

The solution has been successfully moved to Region of Southern Denmark's new integrated and seamless SAS® Platform utilizing the new tools **SAS® Visual Text Analytics**, **SAS® Visual Data Mining and Machine-Learning**, and **SAS® Visual Analytics**. The move to the new and seamlessly integrated analytics platform makes it easier to enhance, maintain, monitor and operationalize the solution.
