# Supplementary material for: Prediction of risk of acquiring urinary tract infection during hospital stay based on machine-learning: A retrospective cohort study
Source: PLoS One. 2021 Mar 31;16(3):e0248636. doi: 10.1371/journal.pone.0248636 (PMC8011767; doi:10.1371/journal.pone.0248636)
Supplement: Attachment — Submitted filename: Response to reviewers.docx [file pone.0248636.s011.docx]

**Response to Reviewers** (PONE-D-20-17945)

5. Review Comments to the Author

Please use the space provided to explain your answers to the questions above. You may also include additional comments for the author, including concerns about dual publication, research ethics, or publication ethics. (Please upload your review as an attachment if it exceeds 20,000 characters)

Reviewer #1: *Healthcare associated infections (HAI), including urinary tract infections (HA-UTI) accounting for about 20-30% of all HAI’s, are a major health care burden. Case finding for the high-risk group of HA-UTI should be instrumental if sufficient sensitivity and specificity can be warranted. There have been previous efforts in a real-time early warning system for monitoring inpatient disease risk, for example PMID 31278734, which should be cited.*

***Response:*** We thank the reviewer for drawing our attention to the interesting and relevant paper of Ye et al. describing “A Real-Time Early Warning System for Monitoring Inpatient Mortality Risk”. We very much agree with the reviewer that a predictive system should be real-time and sufficient sensitive and specific to warrant its clinical use as an early warning system for monitoring inpatient disease risk.

We have made the following additions and changes in the revised manuscript:

i) The paper of Ye et al. (*PMID 31278734)* is cited in the **Introduction** (Page 4, lines 67-70): A real-time early warning system for monitoring inpatient disease risk should reflect underlying clinical causality and thereby enable caregivers to provide clinical intervention or treatment, e.g., in case of inpatient mortality risk [14].

ii) A paragraph referring to the paper of Ye et al. is also added to the **Discussion** (see below for response to reviewer point 3e).

iii) The following sets of amendments and clarifications (in yellow) are added to underscore the point made by the reviewer:

**Abstract** (Page 3, lines 46-50):

*Conclusion*

The study is proof of concept that it is possible to create machine-learning models that can serve as early warning systems to predict patients at risk of acquiring urinary tract infections during admission. The entry model and the HA-UTI models perform with a high ROC-index indicating a sufficient sensitivity and specificity, which may make both models instrumental in individualized prevention of UTI in hospitalized patients.

**5. Conclusion** (Page 22, lines 486-491):
We have shown that it is possible to create machine-learning models that can serve as early warning systems to predict patients in risk of acquiring a urinary tract infection during admission in the Region of Southern Denmark.

Both Models perform with a high ROC-index on the validation data set, Entry Model: 0.81 and HAI Model: 0.74 indicating a sufficient sensitivity and specificity, which potentially makes both models instrumental in individualized prevention of UTI in hospitalized patients.

*However, significant revision should be needed to make this work useful:
1. Data availability – the raw data matrix for each of the data mining modeling (training/testing) should be made available for download so that others can take a spin to replica their work or improve their work.*

***Response:*** The de-identified raw data matrix used is now submitted to the Dryad Digital Repository (doi:10.5061/dryad.6djh9w107).

We have therefore replaced the previous statement about the availability of data with the following sentences (Page 5, lines 104-107):

All data were stored on encrypted computers and servers. The dataset used is available in the Dryad Digital Repository (doi:10.5061/dryad.6djh9w107). Data files contain no sensitive or personally identifiable information.

2. *Machine learning approach – it is great that the authors tried multiple machine learning approaches. But the goal here is not to instruct the audience as how-to cherry pick methods but rather whether we can deliver a HA-UTI model for care management. These can be presented in the appendix if the readers have interest to review and learn.*

***Response:*** We agree that some machine learning details can better be presented in the appendix, e.g. the former Table 3 and Fig 3, which are replaced in the revised manuscript as supporting information: Table S4 and Fig S2.
However, we find it useful for readers to read the “Receiver operating characteristic (ROC) curves for the machine learning models” (Fig 4) and “The cumulative lift for the entry model” (Fig 5) in the main text.

*3. Model and model features – the most effective model should be presented in detail:*
 *a. How were the predictive features selected?*

***Response:*** The predictor features were primarily selected using a literature review of known risk factors as mentioned on page 4 (Introduction) in the primary manuscript. Some candidate variables have furthermore been examined in a previous publication “Analysing risk factors for urinary tract infection based on registrations in our hospital electronic patient records and an automated, routine monitoring of hospital-acquired infections” (Referenced as #15 in the primary manuscript: Redder et al. J Hosp Infect. 2016 Apr;92(4):397-400. doi: 10.1016/j.jhin.2015.12.009.).

i. We have underlined this (yellow) by an addition to the existing description on page 6, lines 131-132:
   Risk factor variables chosen were based upon review of the literature [15] and our previous study on risk factors for urinary tract infection [16].
ii. We have further pinpointed the importance of the issue by adding a new sentence on page 10, section “2.4.1 Variables”, lines 184-187:
    In-put variables were primarily chosen to reflect possible underlying clinical causality, e.g., documented instrumentation of the urinary tract, urine retention diagnosed, presence of Indwelling Urinary Catheter, or previous UTIs detected.
iii. Selected input variables present in the Regional Data Warehouse were scrutinized before use to determine underlying causality as predictor. For example, data collected after detection of a HA-UTI was consequently discarded.
     Furthermore, a multivariate filtering process excluded predictor variables without discriminative impact in the two models examined, see revised version of the former supporting Table S6 now presented in the main text as Table 4 (see also response below to reviewer comment 3b).

b. *The predictive features should be presented with univariate analysis including but not limiting to odds ratio, p value etc.*

***Response:*** We have added the following table to the main text (Page 16, lines 321-325):

**Table 4. Univariate and multivariate analysis of the impactful predictive feature in the Entry-model**.

|  |  | **Analysis of Maximum Likelihood Estimates** | | | |
| --- | --- | --- | --- | --- | --- |
| Predictive feature | Odds  Ratio | DF | Estimate | Standard  Error | Wald  Chi-Square |
| ADMISSION_TYPE (Acute vs Planned) | 2.286 | 1 | 0.4134 | 0.0210 | 388.04 |
| Sex (Female vs Male) | 1.620 | 1 | 0.2412 | 0.0112 | 459.85 |
| ICD-10 COPD^1^, admission (0 vs 1) | 1.244 | 1 | 0.1093 | 0.0236 | 21.38 |
| ICD-10 Neuro Disease, admission (0 vs 1) | 0.725 | 1 | -0.1606 | 0.0255 | 39.79 |
| ICD-10 Diabetes, admission (0 vs 1) | 0.789 | 1 | -0.1184 | 0.0226 | 27.56 |
| ICD-10 Urinary retention, admission (0 vs 1) | 0.496 | 1 | -0.3506 | 0.0382 | 84.10 |
| Age_admission | 1.040 | 1 | 0.0395 | 0.000745 | 2811.38 |
| Previous CAI-UTI (0 vs 1) | 1.748 | 1 | 0.5586 | 0.0238 | 550.17 |
| Previous HAI-UTI (0 vs 1) | 1.153 | 1 | 0.1421 | 0.0363 | 15.29 |
| Previous IUC^2^ (0 vs 1) | 1.178 | 1 | 0.1642 | 0.0186 | 77.66 |
| Readmission (0 vs 1) | 1.167 | 1 | 0.0772 | 0.0185 | 17.47 |

CA-UTI: Community Acquired UTI; HA-UTI: Hospital Acquired UTI.
^1^Chronic obstructive pulmonary disease

^2^IUC: Indwelling urinary catheter

Table 4 is referred to in the main text on page 15, lines 308-311:

As indicated by the number of splitting rules for the various input variables in the entry-model (Table 3), the univariate and multivariate analyses of the risk factors included in the study show that admission type (acute admission), age at admission, sex, and past CA-UTI are impactful predictive features in the model (Table 4).

c. *Currently the feature table is hidden as supplementary. It should be presented in the main text. From the table, looks like the past UTI is the major driver.*

***Response:*** A new Table 3 now replaces the former feature table S6 in the main text under “Results” (page 15, lines 313-319).

We have furthermore added the following paragraph to precede Table 3 on page 15, lines 306-308:

The variable importance table (Table 3) shows the predictor variables that the Decision Tree machine-learning model used for the entry-model. For the HA-UTI model a similar variable importance table (S6) was created.

*d. Should the first time UTI and the repeated UTI modeling efforts be separated as two different learning processes?*

***Response:*** The reviewer’s question/proposal on separating *first time UTI and the repeated UTI modeling* is not quite clear to us. We do not regard first-time UTI and repeated UTI’s as topics for two different learning processes but merely as quantitative aspects of the same risk factor (previous UTI).

We describe two different learning processes: 1) an entry-model using static data available at the time of admission (T=0), and 2) a HA-UTI model using both static data and a set of observational data (T>=48). In the manuscript, we have chosen to focus on the entry-model.
This has now been underscored in the main text on page 13, lines 265-266:

We choose to focus primarily on the entry-model in the presentation and evaluation of results in the present study.

e. *Throughout the paper, there is no discussion of the underlying clinical causality in regard to the driving features? This is a major limitation. Not only we need to predict but also interpret.*

***Response:*** We are grateful for this comment and completely agree with the reviewer that a discussion of the underlying clinical causality between the predictive factors and the outcome variable UTI is mandatory. The overarching objective of our modelling efforts has also been to create an early warning system, where the driving features in the predictive models should represent an early notification of modifiable risk factors providing individualized countermeasures for prevention of UTI in hospitalized patients as a supplement to the general infections control practiced in a hospital.

To underscore the importance of an underlying causality between the driving features of the models (predictive factors) and the development of UTIs, the following additions to the current text have been made:

i: on page 17, lines 350-353:

It is important that an early warning system for monitoring inpatient disease risk factors not only reflects underlying clinical causality but also provides useful information in real-time that enables caregivers to initiate clinical intervention or treatment to prevent or modify the predicted disease event as early as possible [14].

Ii: A supplementary paragraph on the importance of underlying clinical causality of driving features in the predictive models is furthermore inserted in front of the present section of the discussion, where the importance of finding modifiable risk factors for clinical use of the models is underscored on page 19, lines 406-413:

It is important that the driving features in the machine learning models represent a direct causal relation to the outcome variable (UTI) promoting an elimination or modification of a risk factor, e.g. by reducing unnecessary use or improving management of indwelling urinary catheters in high-risk patients. Girard et al. identified the factors associated with HA-UTI in the general population of patients in geriatric hospitals, the most important of which were UTI in the six months before the study, and intermittent bladder catheterization (22). We also found a strong correlation to previous UTIs in a general hospital population, which is likely to be associated with underlying urological pathologies in the patient.

*Therefore, major revision is requested.*

As indicated above we have introduced substantial changes/additions to the main text, tables, and figures of the manuscript in accordance with the response to the reviewer’s comments.

Apart from renaming and moving a former Figure 3 and Table3 to Supporting information, we have added two new tables (3 and 4) to the main text (all marked in yellow).

Two additional literature references are included to support the discussion on the importance of underlying clinical causality of driving features in the predictive models (marked in yellow).
A number of minor linguistic changes (e.g. breaking up a couple of long sentences and deletion of redundant text) of the main text have been made to improve reading of the manuscript (these changes have not been marked in manuscript).
